# No evidence of immunosurveillance in mutation-hotspot-driven clonal hematopoiesis

**DOI:** 10.1038/s41588-026-02594-y

**Published:** 2026-06-29

**Authors:** Barbara Walkowiak, Hamish A. J. MacGregor, Jamie R. Blundell

**Affiliations:** 1 Early Cancer Institute, University of Cambridge, Cambridge, UK; 2 Centre for Cancer Genetic Epidemiology, Department of Public Health and Primary Care, University of Cambridge, Cambridge, UK

**Keywords:** Haematological cancer, Computational biology and bioinformatics, Immunosurveillance

## Abstract

The theory of immunosurveillance posits that T cells eliminate clones harboring nonself-antigens generated by somatic mutations. Although a role of immunosurveillance in cancer is supported by evidence, whether it affects precancerous expansions has not been well established. Here we studied the association between MHC–variant binding and risk of clonal hematopoiesis (CH), a blood precancer state, predicting MHC binding affinity toward CH hotspot variants in 380,000 UK Biobank participants and examining the relationship between predicted binding to each variant and its expansion risk. Despite the study being powered to detect subtle differences in selective pressure, we did not find associations between predicted binding and CH prevalence for any of the examined variants. In CH-affected individuals, we identified no relationship between predicted variant binding and clone size. Overall, we found no evidence that the MHC genotype affects which variants expand in CH, suggesting a limited role for immunosurveillance in shaping clonal expansions in the blood.

## Abstract (web summary)

Analysis of the UK Biobank cohort demonstrates that there is no significant MHC-I- or MHC-II-driven negative selection against expansion of mutant clones in clonal hematopoiesis.

## Main

According to the theory of immunosurveillance, the immune system can identify and eliminate cells carrying somatic mutations, thereby preventing potentially cancerous clones from expanding^1^. All cells display peptides generated from their proteome on their surface, allowing the immune system to detect cells that present nonself peptides. Peptide presentation requires binding to the class I major histocompatibility complex (MHC-I). The resulting peptide–MHC (p-MHC) complex is trafficked to the cell surface, where it can be recognized by CD8^+^ T cells^2^. Whether a given peptide is presented on the surface is influenced by its binding affinity to the MHC, with strongly binding peptides more likely to form the p-MHC complex^3^. MHC alleles are highly polymorphic in the human population^4^; therefore, a peptide may be differentially presented among individuals who carry different MHC alleles. It has been suggested that this may influence the expansion of driver mutations in cancer. Cancers reportedly carry driver mutations that are anomalously poor binders to the patient’s MHC^5^, and particular MHC alleles predicted to bind to mutant peptides with high affinity are underrepresented in some cancers^6^. In addition, recognition by CD8^+^ T cells of neoantigens presented on the MHC-II complex, which is mainly expressed in hematopoietic and immune cells^7,8^, can contribute to detection and elimination of cancer clones^9,10^.

Although immunosurveillance of somatic clones has been extensively studied in the context of cancer^11–15^, it is not fully understood when it begins and to what extent it shapes somatic evolution in aging precancerous tissues. Incidence of cancer increases in immunocompromised individuals^16^, suggesting that the immune system can prevent early clonal expansions from transforming into clinically observable disease. Although no signatures of negative selection or immune system activity were identified in healthy somatic tissues bearing a substantial burden of mutations^17,18^, tissues in early precancer stages have shown signs of immune infiltration^19^. This could be related to the observation that immune system engagement may be dependent on reaching a minimum fraction of cells that bear the neoantigen in the tissue^20^. However, study of early immune surveillance has been challenging owing to small sample sizes and a focus on small clonal expansions. Therefore, datasets in which a large number of clonal expansions can be measured quantitatively are needed to comprehensively address the problem.

We reasoned that the role of immune surveillance during somatic evolution could be investigated by assessing whether the evolutionary trajectory of a variant was influenced by the capacity of the immune system to recognize it. We examined this in CH, a precancer state that results from expansion of hematopoietic stem cells (HS cells) and is associated with increased risk of hematological malignancies^21,22^. CH can be identified in blood exome sequencing data, which is available for cohorts such as the UK Biobank; this enables comparison of expansions of a specific driver hotspot mutation across a large number of individuals who differ with respect to their capacity to present this mutation, providing us with considerable power to detect potential associations. In particular, in individuals whose MHC presents a given variant, we may expect expansions of clones carrying this variant to be more limited. By contrast, in individuals whose MHC does not bind to peptides with this variant, there will be no immune recognition, and so expansions will not be restricted. At the population level, individuals who present a given mutation well are therefore predicted to have an overall lower prevalence of clones harboring this variant.

## Results

### Association between MHC binding and CH prevalence

**Fig. 1:**
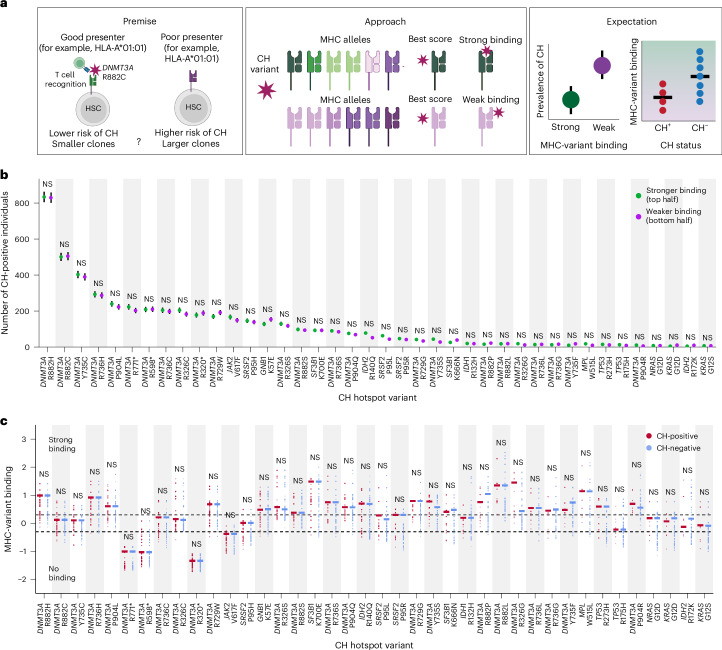
No effect of predicted MHC binding on CH prevalence. **a**, Overview of study hypothesis and approach. **b**, Numbers of CH-positive individuals did not differ among groups of varying relative MHC–variant binding. *P* values are from two-sided Fisher’s exact test (Bonferroni-corrected); error bars represent 95% confidence intervals. **c**, The distribution of MHC–variant binding scores did not differ between individuals who were positive and negative for each variant. For visualization purposes, we compared the scores of all CH-positive individuals to 2,000 randomly sampled individuals who were identified as CH-negative for the variant. *P* values are from two-sided Mann–Whitney *U*-test (Bonferroni-corrected); dashed lines indicate the threshold for strong binding (top line, corresponding to percentage elution rank 0.5) and weak binding (bottom line, percentage elution rank 2). NS, not significant. Source data

The UK Biobank contains whole-exome sequencing data from around 450,000 people between the ages of 40 and 70 years. Owing to the depth of sequencing (median depth of 50×) and known the propensity of CH to occur at certain positions in the genome (CH ‘hotspots’), somatic clones larger than ~2–3% variant allele frequency (VAF) can be robustly identified in these data, allowing us to explore the potential relationship between MHC presentation and CH at considerable scale. If immunosurveillance were able to exert negative selective pressure on somatic clones, we reasoned that individuals predicted to have stronger MHC binding to a specific variant peptide might be at lower risk of developing CH driven by that variant (Fig. 1a). To test this, we obtained imputed MHC genotypes from cancer-free individuals in the UK Biobank^23^ (MHC class I: *n* = 384,600; class II: *n* = 327,961) and used NetMHCpan-4.1^24^ to make computational predictions of MHC-I binding affinity between each individual’s MHC-I alleles and peptides carrying 40 recurrent missense and truncating variants in 11 common CH driver genes^25^ (see Supplementary Table 1 for variant calls and numbers of individuals, and Supplementary Fig. 1). Each person–variant pair was assigned a single MHC binding score defined as the strongest-binding peptide that covered the variant, as measured via elution percentage rank score across all MHC alleles present in that individual (Methods).

**Extended Data Fig. 1:**
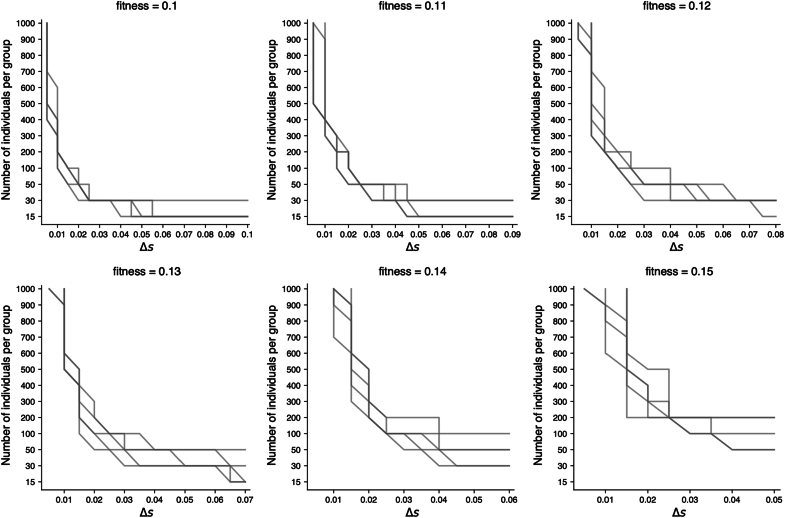
Power analysis for Fisher’s exact test. Results of simulations to determine extent of power to detect the difference in the Fisher’s exact test. Grey lines indicate the boundary between significant and non-significant results for independent sampling of number of individuals. Related to Fig. 1b.

We identified 9,309 individuals who carried a somatic variant in one or more of the hotspot positions using the UK Biobank exome sequencing dataset. First, we split the cohort into two equal-sized groups based on relative predicted binding capacity and compared the number of CH carriers between the stronger- and weaker-binding groups (Fig. 1b and Supplementary Fig. 1). There was no significant difference in CH prevalence between stronger- and weaker-binding individuals for any of the variants studied. This analysis was powered to detect negative selective pressures between 0.5% and 3.0% depending on the fitness of the variant and how many times it was detected (Extended Data Fig. 1).

The same relationship could also be examined by determining whether the distribution of binding cores for a given variant differed between CH carriers and noncarriers. If MHC presentation prevented some variants from expanding, there would be an expectation that binding scores for individuals with expanded clones would fall toward the lower end of scores observed in the general population (Fig. 1a). We compared the distributions of scores between individuals positive and negative for each variant, noting that any clone at sufficiently high VAF to be observable in the UK Biobank must have undergone a period of strong positive selection^25^ (Fig. 1c). Again, we found no significant differences in predicted binding score between CH carriers and noncarriers across the 40 hotspots.

**Extended Data Fig. 2:**
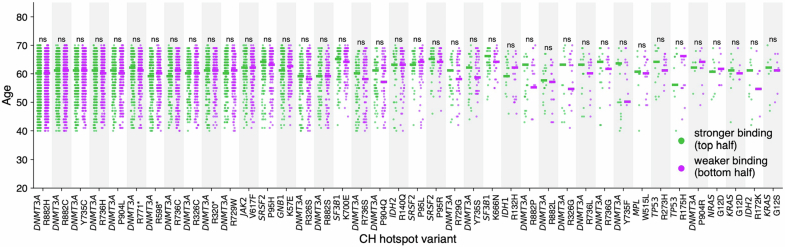
Distribution of ages between carriers in top vs bottom half of the population by binding scores. Ages for stronger binders (green) vs weaker binders (purple) show no significant differences. Source data

**Extended Data Fig. 3:**
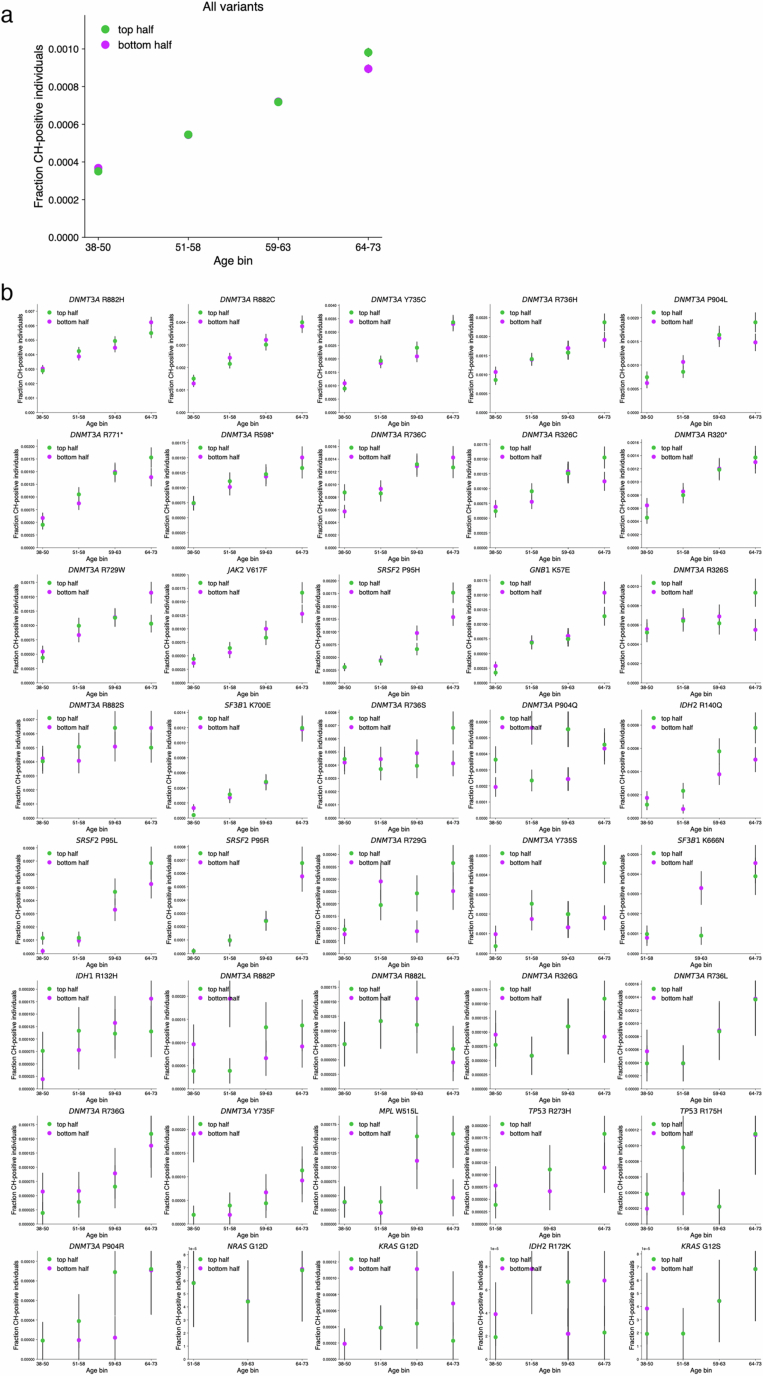
Fraction of CH-positive individuals who are predicted to bind a variant better (’top half’) or worse (’bottom half’) in different age bins. Age bins selected such that the total numbers of individuals in different age bins are balanced. **a**. All variants aggregated together. **b**. Relationship between age and CH prevalence of each individual variant investigated in the study.

The prevalence of CH and size of expanded clones increases with age; therefore, we considered whether there might be evidence of an association between predicted binding and the age distribution of CH-positive individuals. However, we found no systematic differences in the age distribution of CH-positive individuals predicted to have better or worse binding of the variant, and the prevalence of CH did not differ significantly between such individuals within each age bin (Extended Data Figs. 2 and 3).

**Fig. 2:**
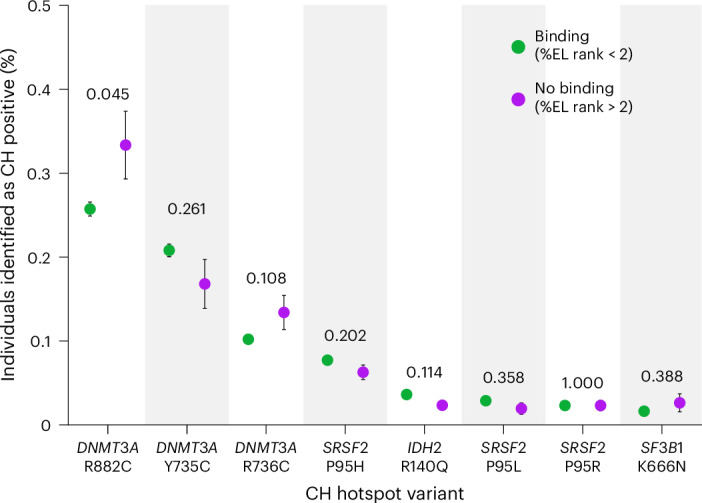
Effect of absolute binding on CH prevalence. Fractions of individuals identified as CH positive between groups of different absolute bindings for the eight variants for which sufficient numbers of individuals carrying the variant were predicted to be strong binders (green data points) or nonbinders (purple data points). Error bars represent 95% confidence intervals. *P* values are from two-sided chi-squared test (Bonferroni-corrected). %EL rank, percentage elution rank. Source data

If binding to MHC required affinities above a specific absolute threshold, examination of absolute binding scores rather than relative binding could have been a more effective way of detecting negative selection. We therefore sorted individuals using absolute (rather than relative) predicted binding score. For the eight hotspots for which at least five individuals carrying the variant were predicted to show strong binding (percentage elution rank < 0.5) and at least five were predicted to have no binding at all (percentage elution rank > 2), we compared CH prevalence between binding and nonbinding individuals (Fig. 2 and Supplementary Fig. 3).

**Extended Data Fig. 4:**
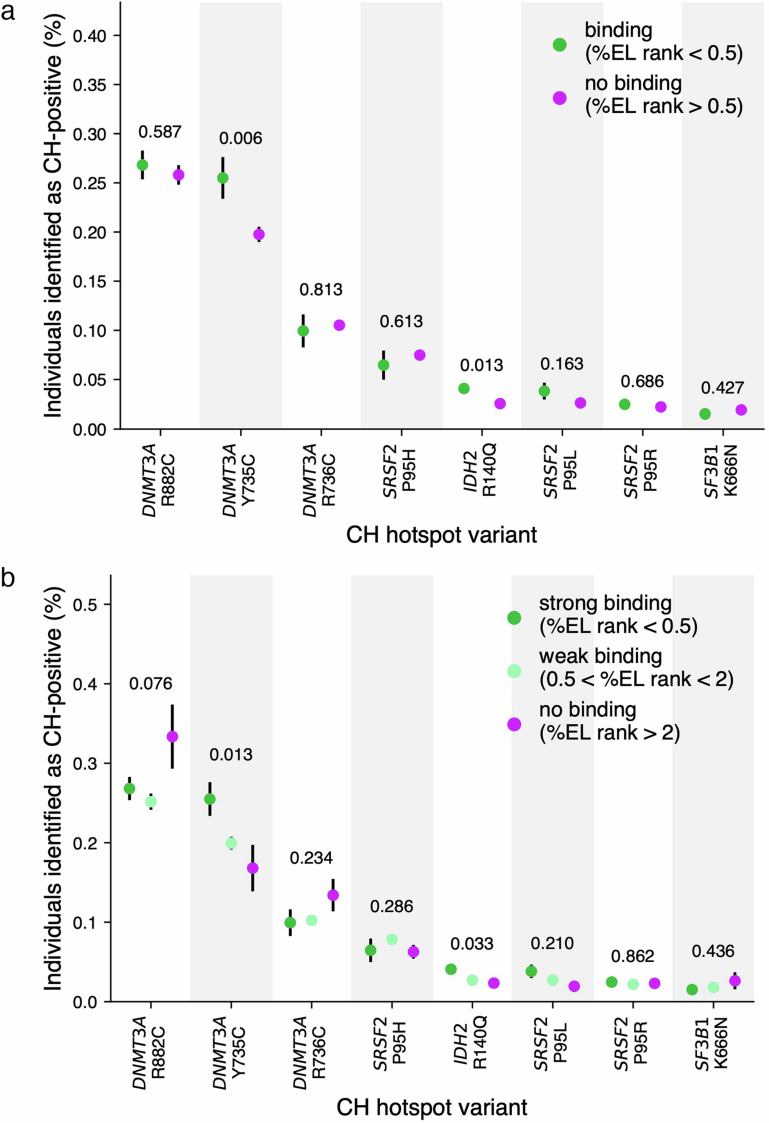
Effect of MHC-variant absolute binding on CH prevalence. **a**. Comparison of the fraction of CH-positive individuals present in groups predicted to bind vs not bind the variant. **b**. Comparison of the fraction of CH-positive individuals present in groups predicted to bind the variant strongly, weakly vs does not bind it. p-value from chi-squared (two-sided) test. Related to Fig. 2. Source data

For seven of the eight variants tested, there was no significant difference in prevalence by predicted binding strength. Only *DNMT3A* R882C-mutant clones showed a marginally significant increase in prevalence in individuals with weaker binding (*P* = 0.045, Bonferroni-corrected). However, this result was not robust to changes in the binding threshold (Extended Data Fig. 4). These findings suggest again that MHC genotype does not influence clonal expansions driven by these variants.

Recent studies have proposed an association between greater diversity in an individual’s MHC-I and MHC-II alleles and protection against lung cancer^26^ and some subtypes of lymphoma^27^. Individuals with a more diverse MHC can present a greater diversity of mutant peptides, potentially conferring better protection against expansion of a range of mutant clones. Therefore, we tested whether the number of MHC alleles and MHC heterozygosity status influenced CH risk. However, we found no association between greater diversity of MHC alleles and decreased prevalence of CH (Supplementary Fig. 4).

### Clone sizes are unaffected by MHC binding

**Fig. 3:**
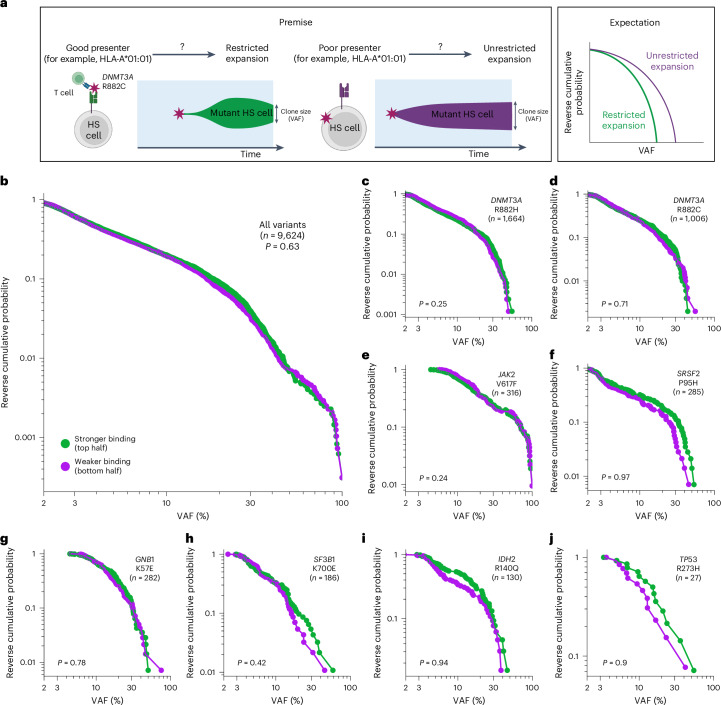
No effect of predicted relative MHC binding on clone size distribution. **a**, Premise and expectation. **b**, The distribution of clone sizes was not associated with differences in clone sizes across all variants. **c**–**j**, Relationship between predicted binding capacity for several representative CH variants: *DNMT3A* R882H (**c**), *DNMT3A* R882C (**d**), *JAK2* V617F (**e**), *SRSF2* P95H (**f**) *GNB1* K57E (**g**), *SF3B1* K700E (**h**), *IDH2* R140Q (**i**), *TP53* R273H (**j**). *P* values are from Kolmogorov–Smirnov test (one-sided).

**Extended Data Fig. 5:**
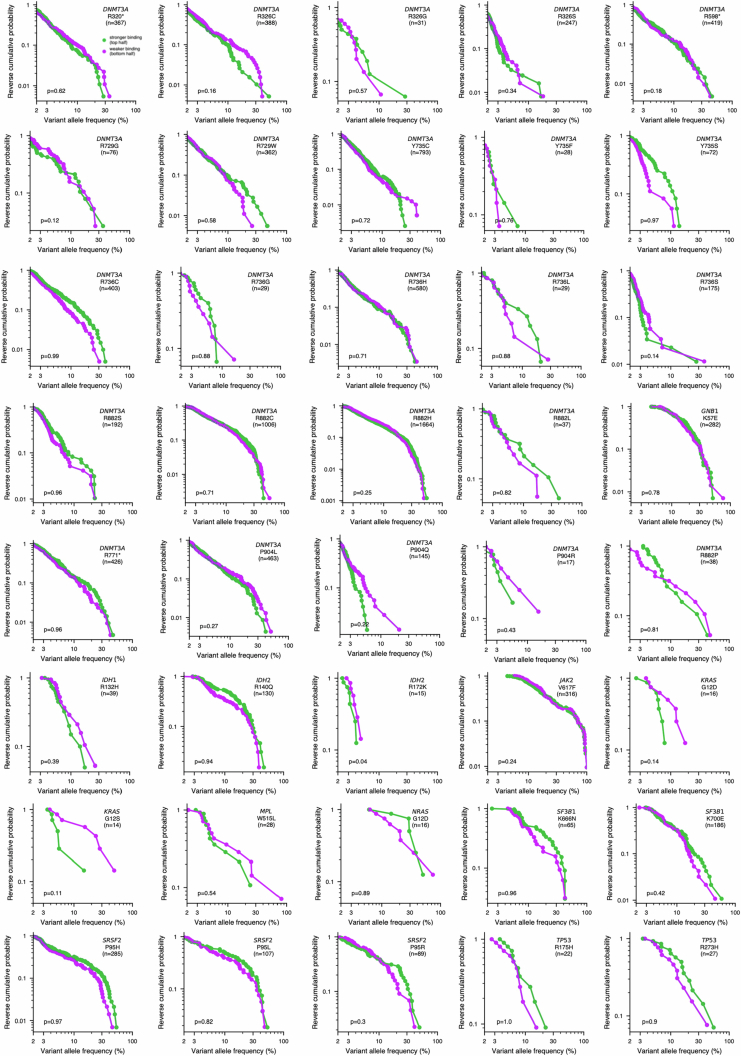
Relationship between predicted MHC-variant binding capacity and clone size in CH-positive individuals for each of the variants examined. p-value from Kolmogorov-Smirnov test (one-sided). Related to Fig. 3.

**Extended Data Fig. 6:**
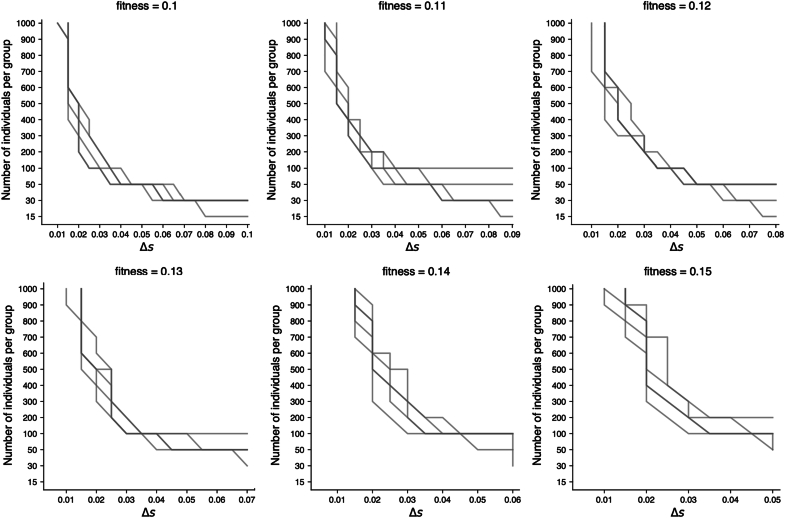
Power analysis for Kolmogorov-Smirnov test. Results of simulation to determine extent of power to detect the difference in clone sizes in Kolmogorov-Smirnov test. Grey lines indicate the boundary between significant and non-significant results for independent simulations. Related to Fig. 3 and Extended Data Fig. 5.

Although the previous analysis suggested that comprehensive immune suppression of clonal expansion of HS cells driven by variants examined here was unlikely to occur in any substantial fraction of the population, we considered whether we might be able to detect more nuanced effects by examining the distribution of clone sizes. If immune surveillance was only initiated once clones reached a certain size, for example, then we would expect the clone size distributions for stronger- and weaker-binding individuals to diverge above a certain clone size (Fig. 3a). The size distributions in CH of expanded clones that we could detect in this study could be accurately estimated using the VAF in peripheral blood. We divided carriers of each variant into equal-sized groups based on relative binding score and compared the distribution of VAFs between the stronger- and weaker-binding groups. We observed no significant difference between the stronger- and weaker-binding VAF distributions, either with all variants aggregated together to increase power (Fig. 3b) or on an individual variant level (Fig. 3c–j and Extended Data Fig. 5). This analysis was powered to detect negative selective pressures of 4% for variants detected in ~100 individuals, 2.5% for variants detected in ~300 individuals and 1% for variants detected in ~1,000 individuals (Extended Data Fig. 6).

**Fig. 4:**
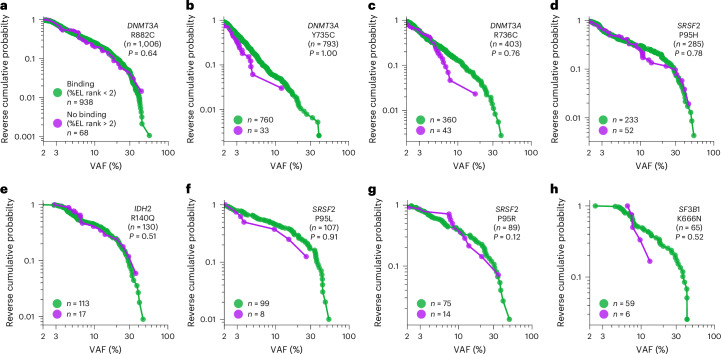
No effect of predicted absolute MHC binding on clone size distribution. **a**–**h**, Reverse cumulative clone size distributions for predicted strong MHC binders (green) and predicted nonbinders (purple) based on absolute binding thresholds: *DNMT3A* R882C (**a**), *DNMT3A* Y735C (**b**), *DNMT3A* R736C (**c**), *SRSF2* P95H (**d**), *IDH2* R140Q (**e**), *SRSF2* P95L (**f**), *SRSF2* P95R (**g**) and *SF3B1*K666N (**h**). *P* values are from Kolmogorov–Smirnov test (one-sided). Source data

**Extended Data Fig. 7:**
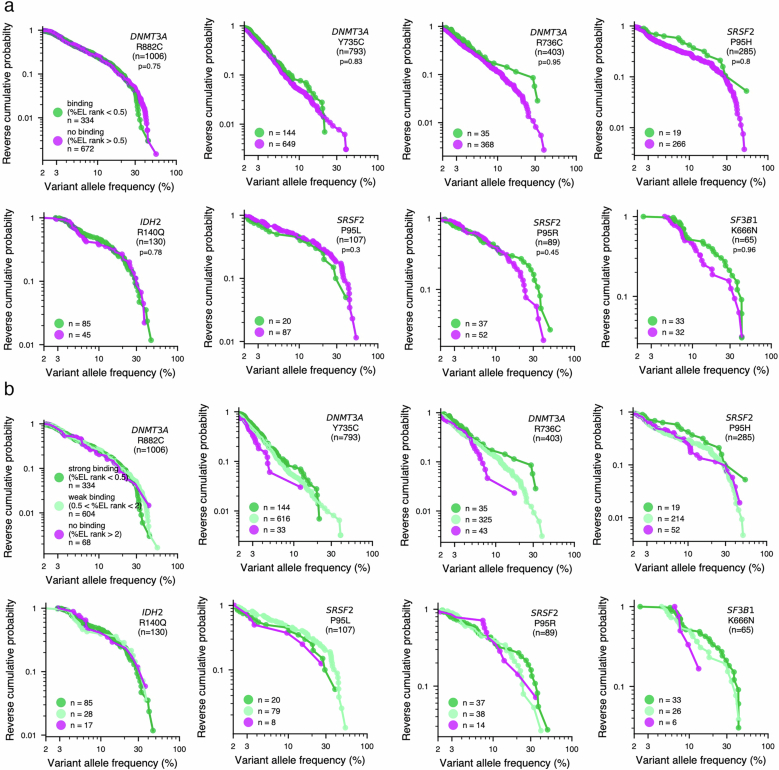
Effect of predicted MHC binding on clone size distribution. **a**,**b**, Absolute binding assessed at threshold of % elution rank 0.5 (strong binding) and at two thresholds (% elution rank 0.5 and % elution rank 2) split into two binding classes (**a**) or three binding classes (**b**). p-value from Kolmogorov-Smirnov test (one-sided). Related to Fig. 4. Source data

As above, we repeated this analysis with the cohort split according to absolute predicted binding score, comparing individuals predicted to show binding of the mutant peptide against those predicted to show no binding at all. We found no significant differences in clone size distributions between these groups (Fig. 4 and Extended Data Fig. 7).

**Extended Data Fig. 8:**
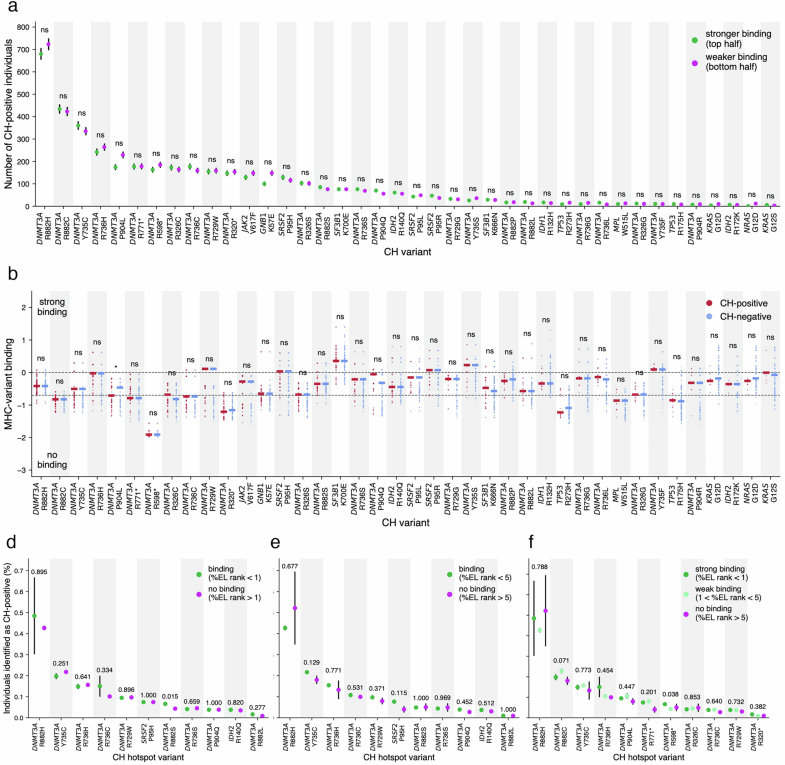
Additional analysis based on binding predictions between CH variants and MHC II alleles. **a**. Comparison of the number of CH-positive individuals between equal-sized groups predicted to bind a given variant better or worse based on their MHC II genotype. p-value from Fisher’s exact test (Bonferroni-corrected). **b**. The distribution of MHC-variant binding scores between CH-positive and 2,000 randomly sampled CH-negative individuals. Variants with at least 5 CH-positive individuals identified as strongly binding the variant and 5 who were predicted not to bind the variant. Dashed lines indicate threshold for strong binding (top line) and weak binding (bottom line). p-value from Mann-Whitney U test (Bonferroni-corrected). Related to Fig. 1. **d***. Fraction* of individuals identified as CH-positive between groups predicted to have differential capacity of variant binding based on MHC II genotype. A. Comparison of the fraction of CH-positive individuals present in groups predicted to bind vs not bind the variant (threshold %EL rank = 5). **e**. Comparison of the fraction of CH-positive individuals present in groups predicted to bind vs not bind the variant (threshold %EL rank = 1). **f**. Comparison of the fraction of CH-positive individuals present in groups predicted to bind the variant strongly (threshold %EL rank = 1.), weakly vs *does not bind* it. p-value from Chi-squared test. Related to Fig. 1*and* Fig. 2. Source data

**Extended Data Fig. 9:**
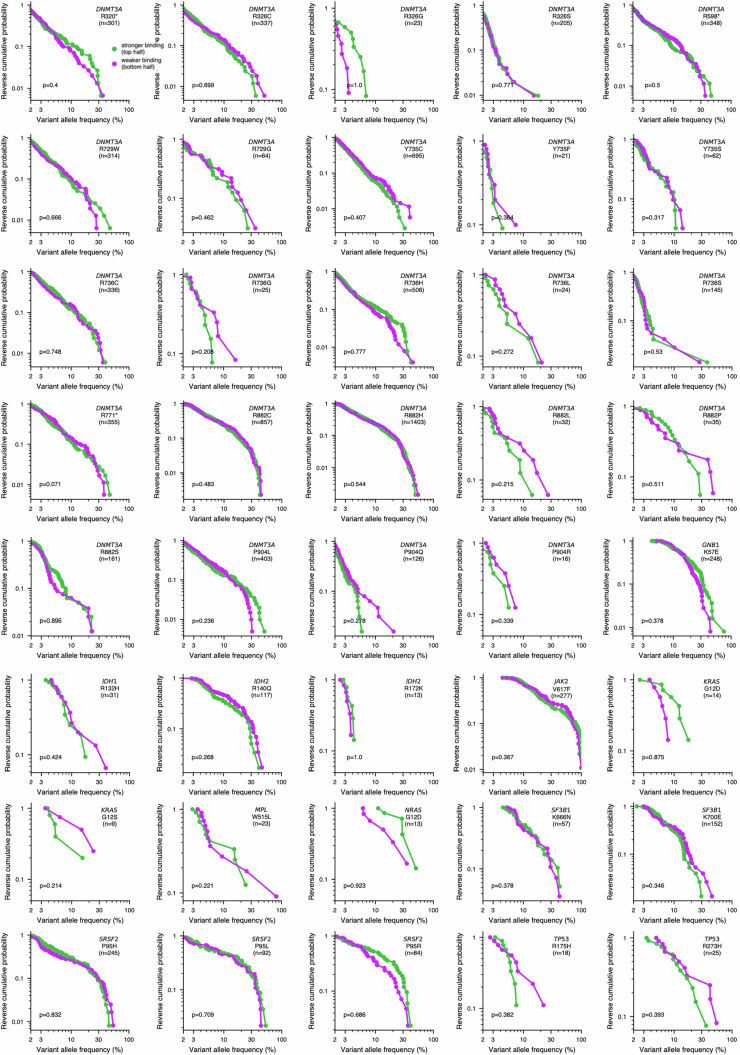
Additional analysis based on binding predictions between CH variants and MHC II alleles. Comparison of reverse cumulative distributions between CH-positive individuals predicted to be in top vs bottom halves of binding each examined variant based on MHC II genotype. p-values from Kolmogorov-Smirnov test (one-sided). Related to Fig. 3. Source data

**Extended Data Fig. 10:**
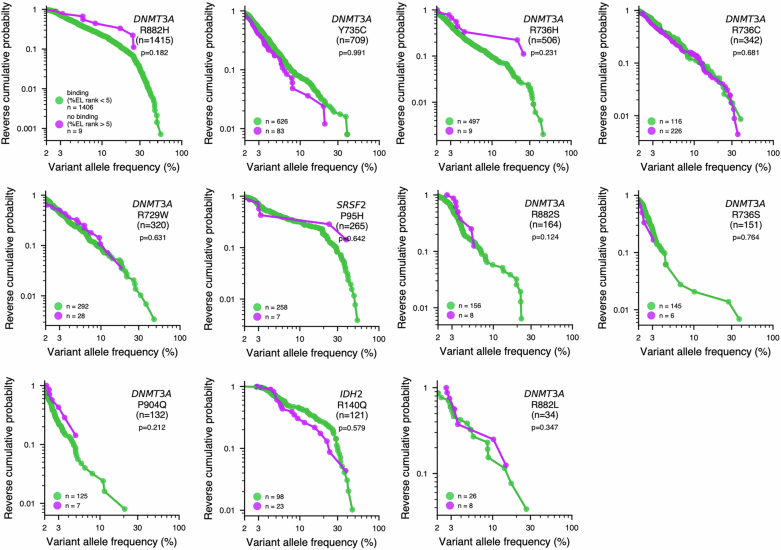
Additional analysis based on binding predictions between CH variants and MHC II alleles. Comparison of reverse cumulative distributions between CH-positive individuals who were classified as binding vs non-binding based on MHC II genotype. p-values from Kolmogorov-Smirnov test (one-sided). Related to Fig. 4. Source data

HS cells have been shown to express MHC-II class alleles^7,28^, and it has been suggested that this could allow surveillance of HS cells by CD4^+^ T cells and elimination of cells carrying mutations that generate immunogenic peptides, providing protection from cancer^6,7^. Therefore, we also tested the association with binding capacity defined by MHC-II genotype, using predictions obtained from NetMHCIIpan-4.3^29^. However, we found no evidence for an association between MHC-II genotype and risk and progression of CH driven by specific variants (Extended Data Figs. 8–10).

Our analysis relied on accurate predictions of MHC–peptide binding; therefore, to mitigate possible biases associated with the specific prediction software, we also used predictions of variant binding to MHC-I from a different method, PRIME2.0^30^. However, in all cases, we reached the same conclusions (Supplementary Figs. 3–6).

### Variants with functionally validated MHC restrictions

**Fig. 5:**
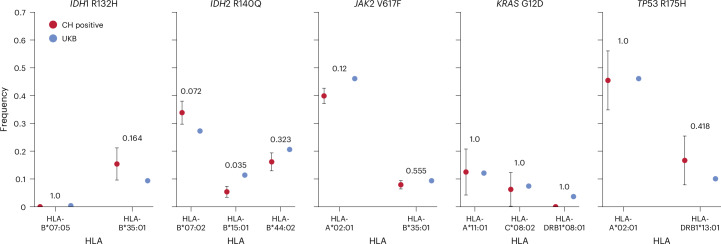
Limited evidence of effect of MHC genotype in MHC-restricted immunogenic variants. Comparison of frequencies of MHC alleles experimentally validated to present specific CH hotspot variants between individuals harboring the variant and the general UK Biobank population. *P* values are from two-sided Fisher’s exact test, and error bars represent 95% confidence intervals. Source data

The reliance on computational prediction of MHC binding represented a limitation of our previous analysis. Given that MHC binding predictions do not directly correspond to immunogenicity, we considered whether we could observe signs of immune surveillance of variants that are known to be MHC restricted (that is, presented only on specific MHC alleles). Were a given variant to be well presented on a specific MHC allele and capable of eliciting an immune response, the expectation would be that this allele would be underrepresented in individuals affected by CH driven by the variant compared to the general population. Therefore, we selected variants for which there was experimental support for MHC restriction (*KRAS* G12D^31,32^, *IDH1* R132H^33^, *IDH2* R140Q^34,35^ and *TP53* R175H^36,37^) and compared the frequency of MHC alleles known to present these variants between the UK Biobank cohort and individuals positive for those variants. We also tested *JAK2* V617F in HLA-B*35:01 and HLA-A*02:01 backgrounds, as these have been previously reported to be underrepresented in patients with myeloproliferative neoplasm carrying the *JAK2* mutation^38^. However, we did not find evidence that these alleles were less common in individuals in whom the variant was expanded than in the UK Biobank cohort (Fig. 5), as would have been expected if MHC presentation led to strong immune surveillance.

## Discussion

Here we have attempted to shed light on the question of whether the immune system exerts negative selective pressures on clones that arise and expand during somatic evolution in aging blood by examining the association between MHC genotype and variants that successfully expand in CH. Although the scale of the UK Biobank affords considerable power to detect weak selective pressures, we found no evidence that MHC-based presentation plays a part in selection of which clones successfully expand in a given individual, arguing against a substantial impact of adaptive immune surveillance on somatic evolution of the blood. We did not detect any difference in predicted MHC binding capacity between individuals who were CH positive or negative for any of the variants examined, nor did we observe differences in proportions of CH-affected individuals across groups of different predicted binding capacity to any variant. Moreover, we did not observe a consistent relationship between predicted binding capacity and the distribution of clone sizes in individuals positive for a given variant. Thus, although absence of evidence does not equate to evidence of absence, our results argue against a strong role for immune surveillance in shaping the somatic diversity of blood in the context of precancerous clonal expansions.

A possible reason that early precancerous clonal expansions do not elicit an immune response could be a requirement for clones carrying nonself-antigens to reach a threshold fraction for T cell recognition^20,39^. For the range of clone sizes examined here, we did not find evidence of a threshold clone size being required for immune recognition in CH, although deeper error-corrected sequencing might be required to detect such effects. In addition, activation of T cells requires prior stimulation of the innate immune system^40^. Small clonal expansions may not generate sufficient signals to trigger innate immune activation, potentially preventing any downstream T cell response.

Although we focused on blood owing to the availability of sufficiently large datasets, it is possible that in epithelial tissues, which can be more inflamed, immune surveillance could occur earlier; this would be an interesting question to explore further. We note that studies of somatic evolution in epithelial tissues have not identified features of an immune response in epithelial tissues despite a substantial burden of mutations in expanding clones^17,18^, and other analyses have suggested that selective pressure exerted by the immune system is stronger in cancer than in precancerous lesions^41,42^.

Our analysis relied on computational predictions of MHC binding. The accuracy of these may be imperfect^43^, and they do not directly correspond to peptide immunogenicity^43–47^; therefore, it is possible that some of the variants examined here were not sufficiently immunogenic to induce an effective immune response even if predicted to be strongly bound by the MHC. This could explain why some other mutations—such as *CALR* frameshifts, which can drive myeloproliferative neoplasms—have been argued to be under early immune surveillance based on the presence of memory T cells and antibodies to CALR in healthy individuals^48^. It is possible that immune surveillance could occur early but only for highly immunogenic mutations, which also seem to trigger an immune response at a lower threshold of clonal fraction^20^. In our study, we did not find evidence of surveillance for variants for which there is experimental evidence of immunogenicity on certain MHC backgrounds, for example, *KRAS* G12D^49^, *TP53* R175H^36^, *IDH2* R140Q^35^ and *IDH1* R132H^33^. However, we cannot exclude the possibility that clones that expand successfully evade immune detection through other mechanisms that could not be established based on the available data alone, such as a reduction in MHC expression or an immunosuppressive environment in the bone marrow. For example, it has been suggested that *JAK2* V617F-positive clones downregulate MHC^38^ and increase expression of PD-L1^50^, which could explain why despite immunogenicity, only minor responses to *JAK2* V617F were found in patients with myeloproliferative neoplasm^51^. Moreover, MHC expression is reduced in maturing HS cells, and hematopoietic cells that differentiate into nonprofessional antigen-presenting cells downregulate MHC-II^7^. Therefore, expansions arising from mutant maturing progenitor cells would be less efficiently detected by the immune system.

Research suggests that mutations that drive clonal expansions in CH in the elderly can be acquired as early as in utero^52–55^. As this developmental stage is associated with a more tolerogenic immune environment^56^, mutations acquired early in life could possibly be tolerated, such that expansions of these clones later in life would not be under surveillance. It is also possible that immune recognition has occurred, but T cells become exhausted through to persistent exposure to the antigen owing to the slow nature of clonal expansions in CH^57^.

Previous studies have suggested that individuals’ predicted MHC binding to mutations present in tumors is poorer than that for mutations absent from the tumors, suggesting that tumor development occurs in the ‘gaps’ in an individual’s immune system^5,9^. We reproduced this analysis for CH by comparing each individual’s predicted binding score for the CH variant they carry (’present’) with their respective scores for the remaining common CH mutations not observed in that individual (’absent’) (Supplementary Fig. 9a). Although the average binding scores for CH carriers varied considerably among variants, we did not observe consistently lower binding scores between the present CH driver and the other possible (absent) mutations. Aggregating these scores across variants can lead to apparent significant differences in the binding capacity of present versus absent variants (Supplementary Fig. 9b). However, this apparent effect is driven predominantly by MHC binding of the most common variants. In this case, this effect was because the most common variants (*DNMT3A* R882 mutations) were also predicted to be bound universally well and would be observed even if all individuals presented the variant equally well^58^. Several CH driver variants we investigated have been predicted to bind different MHC alleles with a similar affinity, such that there is little variation in binding of those variants across the population. In these instances, the effect of the MHC on which variants are allowed to expand may not be as robust as previously suggested^5,9^. The UK Biobank cohort is rather uniform with respect to its ancestry and genetic make-up, and we may see larger variation in MHC binding in more diverse cohorts^59^; furthermore, there may be cases of mutations that generate peptides that are both strongly immunogenic and sufficiently differentially bound between MHC alleles for which such an effect would still be observed^6^, but this may not generalize across all cases of driver mutations.

CH is known to be associated with increased risk of hematological cancers^21,60^ and cardiovascular disease^61^; therefore, limiting expansions of CH clones, for example, through vaccination, would be an attractive strategy to reduce the risk of associated diseases. Although our analysis suggests that immune surveillance does not affect the expansions of most known drivers of CH, it does not exclude the possibility that an immune response could be elicited in this way. Functional studies would be needed to evaluate the possibility of altering the evolutionary trajectory of clones expanded in CH through such interventions.

## Methods

### Statistics and reproducibility

No statistical method was used to predetermine sample size. The analysis was performed on all individuals for whom exome sequencing and MHC genotype data were available (see ‘UK Biobank exome sequencing’ and ‘MHC typing in the UK Biobank’).

### UK Biobank exome sequencing

Exome sequencing of 450,000 people was performed as part of the UK Biobank^62,63^, a prospective cohort study of 500,000 middle-aged people in the UK. All UK Biobank participants signed a written informed consent form at enrollment. Ethical approval was given by the North West Multi-Centre Research Ethics Committee (REC 21/NW/0157).

### MHC typing in the UK Biobank

MHC types were imputed by Bycroft et al. at two-field (four-digit) resolution for 11 classical HLA genes (*HLA-A*, *HLA-B*, *HLA-C*, *HLA-DRB1*, *HLA-DRB3*, *HLA-DRB4*, *HLA-DRB5*, *HLA-DQA1*, *HLA-DQB1*, *HLA-DPA1* and *HLA-DPB1*) using the HLA*IMP:02 algorithm with a multipopulation reference panel^23^. The MHC genotyping dataset contains posterior probability values for each MHC allele (0, not present; 1, one allele present; 2, both alleles present). According to published recommendations^64^, a threshold of 0.7 was used to identify individuals positive for a given allele, and one of 1.5 to identify individuals carrying two copies of a given allele. Individuals were excluded from further analysis if not all of their alleles could be genotyped with confidence, and filtering was done separately for the MHC-I and MHC-II genotypes to avoid exclusion of individuals who only had MHC-I or MHC-II genotype missing.

### Calling CH hotspot variants from UK Biobank exome sequencing

Variants at previously identified CH hotspots were called de novo from UK Biobank exome CRAM files using the DNAnexus Research Analysis Platform (application 28126)^65^. samtools mpileup (docker: quay.io/biocontainers/samtools:1.12–hd5e65b6_0) was used to identify variants at 26 genomic positions in 11 genes, corresponding to common single nucleotide variants identified as potential drivers of CH by Watson et al.^25^ (Supplementary Table 1). As part of a separate analysis, variants in *TP53* were obtained from the same CRAM files using mutect2, a somatic variant caller available in the Genome Analysis Toolkit (docker: broadinstitute/gatk:4.1.3.0)^66^. We restricted our analysis to variants that were present in more than 15 individuals in the entire UK Biobank cohort. The number of unique variants analyzed (after all filters were applied) was 40.

To remove false positives caused by sequencing errors, variants not supported by at least two variant reads were discarded (three in *TP53*). Individuals carrying a mosaic chromosomal alteration overlapping the variant (as identified by Loh et al.^67^) were excluded. As certain types of systemic cancer treatment are known to alter the selection landscape of CH^68^, individuals with a history of cancer before blood draw were excluded, including those with self-reported cancers (with the exception of basal cell carcinoma, precancer of the cervix and rodent ulcer).

After the CH and MHC filtering steps, there were 384,600 individuals with MHC-I allele data available, of whom 9,309 carried at least one CH variant (9,624 variants). For MHC-II, there were 327,961 individuals and 7,932 individuals (8,212 variants).

### MHC–variant binding predictions

The genomic coordinates of CH variants were obtained from COSMIC^69^. Corresponding amino acid sequences were obtained from the UniProt database (UniProt Consortium, 2023). We obtained peptide sequences of appropriate length (8–11 for MHC class I and 15 for MHC class II) containing the position where the mutation occurred.

Predictions were generated for all MHC alleles typed at least once in the UK Biobank that were available in NetMHC-pan4.1^24^ or NetMHCIIpan-4.3^29^. These included 194 MHC class I alleles (A type, B type, C type) and 441 MHC class II alleles or allele combinations (DRB1, combinations of DPA-DPB alleles and combinations of DQA-DQB alleles). DRB3, DRB4 and DRB5 alleles were excluded from analysis as they were missing in 50% of the examined population.

To obtain predictions for each variant–allele combination, we first generated binding predictions for each variant-carrying peptide and allele combination and chose the score of the best-scoring peptide as the variant score. Percentage minimum elution rank score was used in the main analysis as it is considered to be the most biologically relevant metric. For each individual, we obtained a single score for each CH variant by identifying the best (lowest) percentage minimum elution rank score across combinations of the gene with all alleles carried by this individual. We obtained two separate scores, one for MHC class I and the other for MHC class II.

### Logistic regression analysis

For all examined individuals in whom MHC genotype was typed with high confidence, we defined MHC heterozygosity status for each locus (MHC class I: A, B, C; MHC class II: DPA, DPB, DQA, DQB, DRB1) and number of distinct MHC-I and MHC-II alleles typed. We also collected data on sex, age at first assessment and smoking status (1, ever smoker; 0, not smoking). Owing to missing data, we restricted our analysis to individuals of European genetic ancestry. For each individual, we defined CH diagnosis as the presence of two or more reads corresponding to at least one CH variant examined here (three in cases of variants in *TP53*). Individuals were excluded from regression analysis if age data were missing.

### Power analysis

To assess the power of our study to detect differences in variant prevalence and VAF distributions between cohorts, we performed simulations using a custom Python script adapted from that of Watson et al.^25^. We simulated clonal dynamics for a range of fitness coefficients (0.1 to 0.2) and sampled different numbers of individuals to approximate the distributions observed in the UK Biobank. At each level of sampling, we determined the minimum difference in fitness effect for which the prevalence was distinguishable using Fisher’s exact test at a significance level of 0.05, and that for which VAF distributions were distinguishable using the Kolmogorov–Smirnov test at a significance level of 0.05. We sampled each number of individuals five times to assess the extent of variability in the power to identify differences between different fitness effects.

### Reporting summary

Further information on research design is available in the Nature Portfolio Reporting Summary linked to this article.

## Online content

Any methods, additional references, Nature Portfolio reporting summaries, source data, extended data, supplementary information, acknowledgements, peer review information; details of author contributions and competing interests; and statements of data and code availability are available at 10.1038/s41588-026-02594-y.

## Supplementary information

Supplementary Information Supplementary Figs. 1–9 and Table 1.

Reporting Summary

## Source data

Source Data Raw source data underlying Figs. 1, 2, 4 and 5 and Extended Data Figs. 2, 4 and 7–10.

## Data Availability

The analysis of UK Biobank data was conducted using the UK Biobank Resource under application number 28126. Source data are provided with this paper.

## Code availability

Code used for the analysis is available via GitHub at https://github.com/the-blundell-lab/MHC_ClonalHaematopoiesis and via Zenodo at 10.5281/zenodo.17348768 (ref. ^70^).
