# Supplementary material for: No evidence of immunosurveillance in mutation-hotspot-driven clonal hematopoiesis
Source: Nat Genet. 2026 Jun 29;58(7):1643–50. doi: 10.1038/s41588-026-02594-y (PMC13364711; doi:10.1038/s41588-026-02594-y)
Supplement: Supplementary file 1 — Supplementary Figs. 1–9 and Table 1. [file 41588_2026_2594_MOESM1_ESM.pdf]

nature genetics

Supplementary information https://doi.org/10.1038/s41588-026-02594-y

No evidence of immunosurveillance in mutation-hotspot-driven clonal hematopoiesis

In the format provided by the authors and unedited

Supplementary information for NG-A66766R1

Supplementary figures

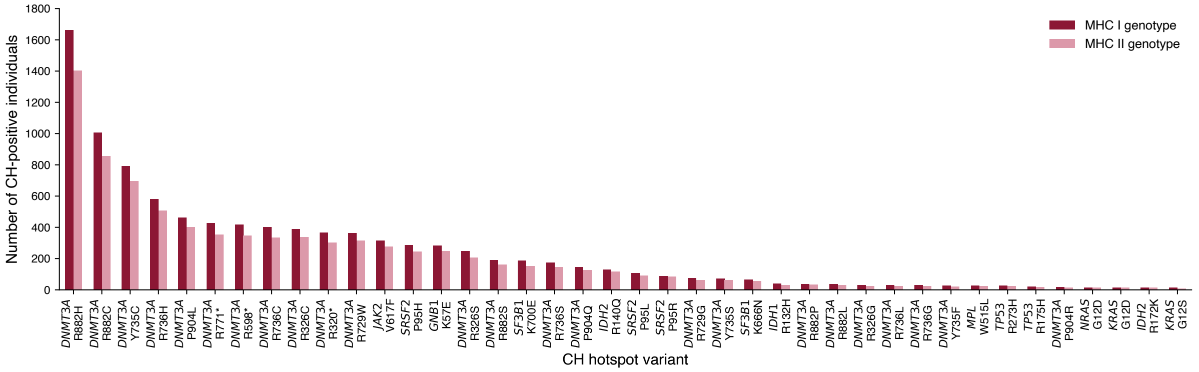

Supplementary Figure 1. Number of individuals carrying each CH hotspot variant for whom we were able to confidently genotype MHC I / MHC II class alleles.

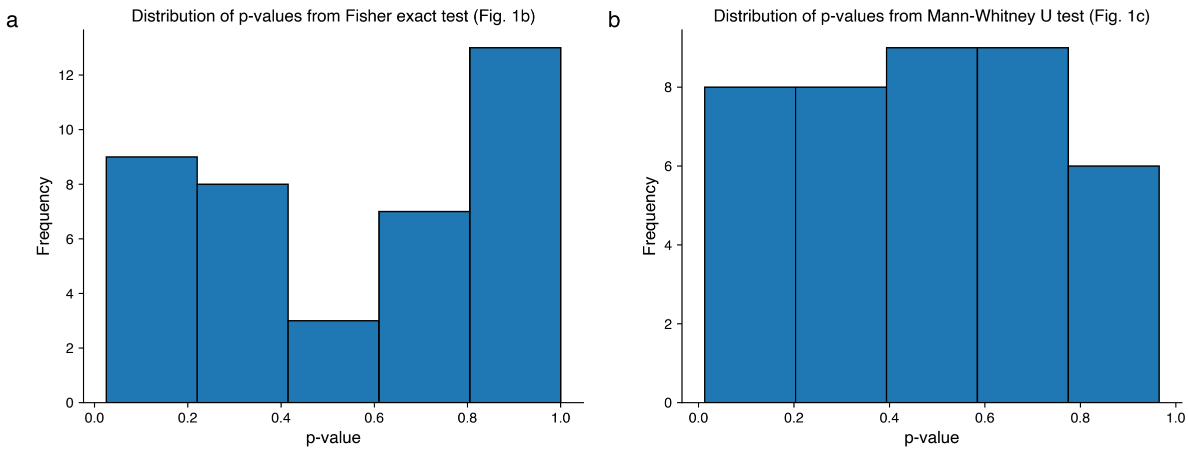

Supplementary Figure 2. Statistical analysis **a**. Distribution of p-values from Fisher's exact test conducted on Fig. 1b to analyze the differences in the number of CH-positive individuals between better and worse binding groups for each CH variant. **b**. Distribution of p-values from Mann Whitney U test conducted on Fig. 1c to compare the MHC-variant binding scores between CH-positive and CH-negative individuals.

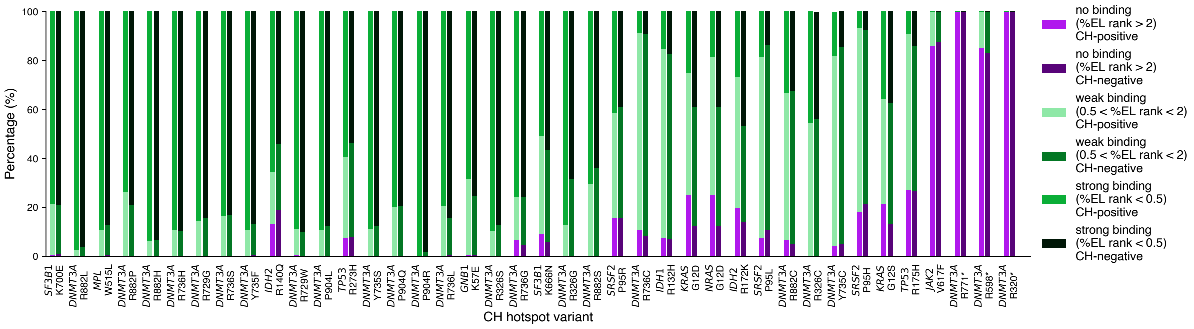

Supplementary Figure 3. No difference in percentage of the population predicted to bind a given variant between CH-positive and CH-negative individuals. Distribution of individuals who are predicted to bind each variant strongly, weakly or not based on their MHC I genotype, in CH-positive and CH-negative individuals for each variant.

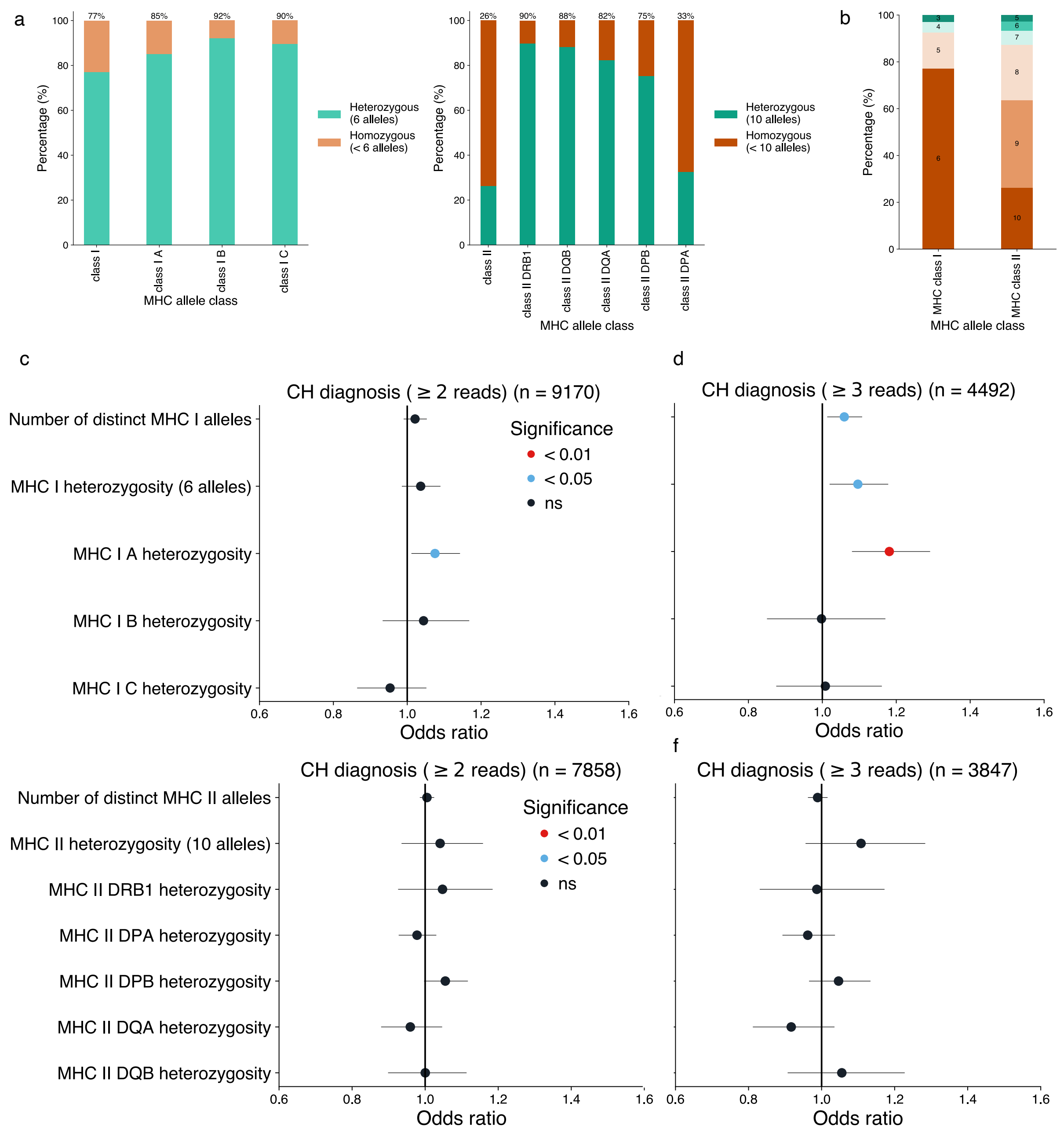

Supplementary Figure 4. No impact of heterozygosity status on overall risk of CH. **a**. Distribution of individuals who are heterozygous vs homozygous for each major class of MHC I or MHC II alleles in the UKB cohort. **b**. Distribution of the number of MHC I and MHC II alleles in the UKB cohort. **c-d**. Relationship between number of MHC I alleles, MHC I heterozygosity status (overall vs specific alleles) and CH diagnosis with threshold of 2 (c) or 3 (d) reads. **e-f**. Relationship between number of MHC I alleles, MHC I heterozygosity status (overall vs specific alleles) and CH diagnosis with threshold of 2 (e) or 3 (f) reads.

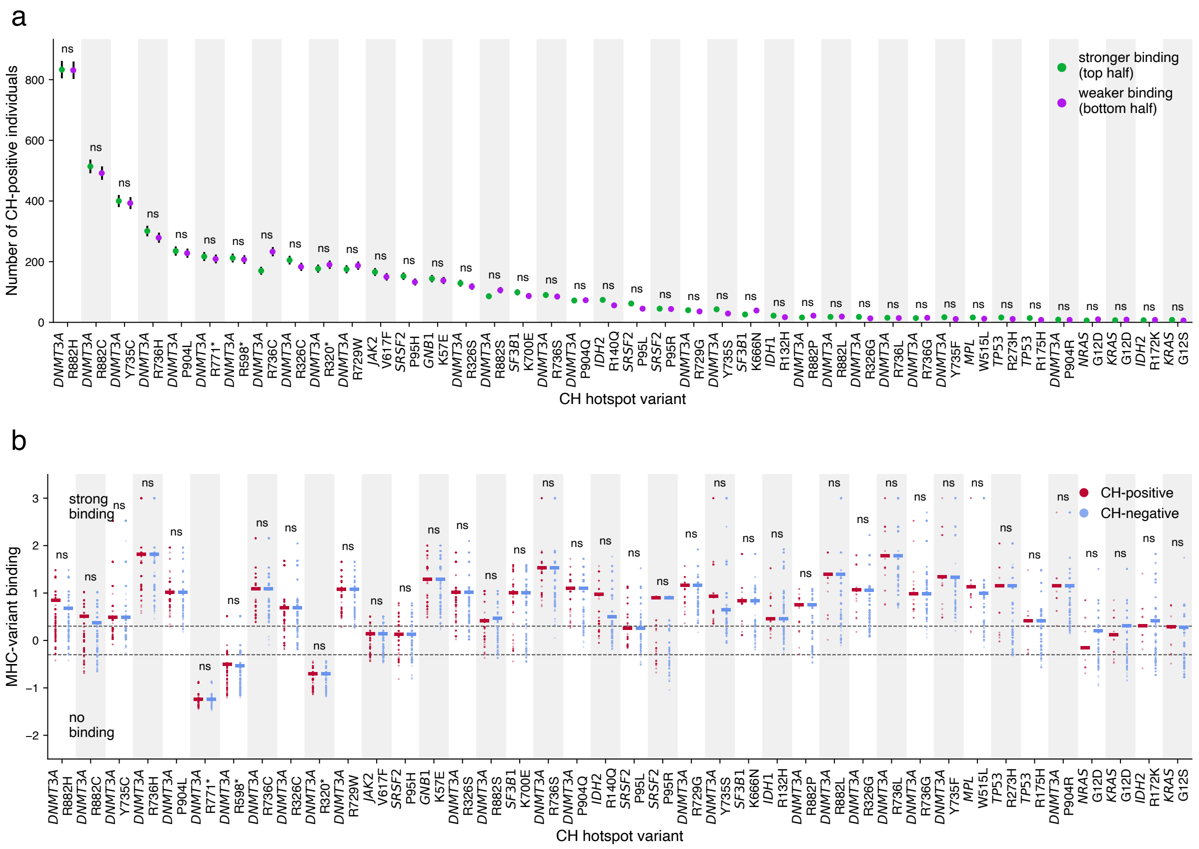

Supplementary Figure 5. Additional analysis based on binding predictions from PRIME2.0. **a**. Comparison of the number of CH-positive individuals between equal-sized groups predicted to bind a given variant better or worse. **b.** The distribution of MHC-variant binding scores between CH-positive and 2,000 randomly sampled CH-negative individuals. Dashed lines indicate threshold for strong binding (top line) and weak binding (bottom line). ns - not significant. Related to Fig. 1.

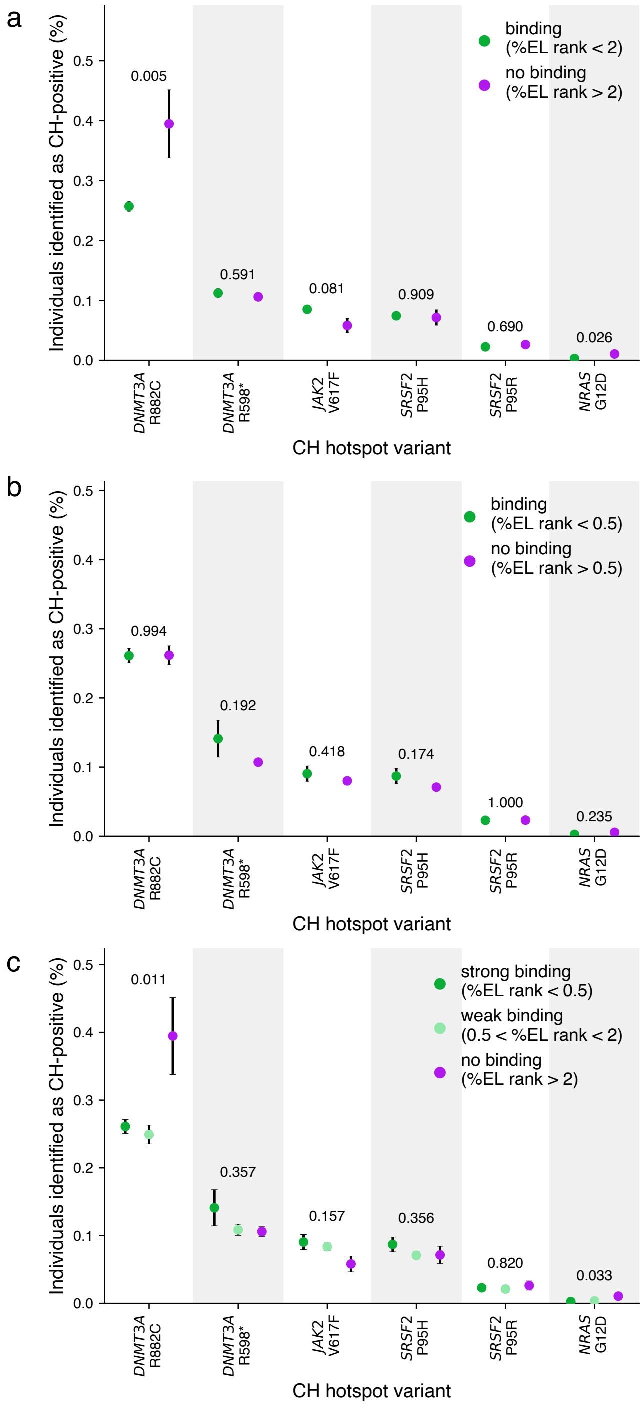

Supplementary Figure 6. Additional analysis based on binding predictions from PRIME2.0 **a**. Comparison of the fraction of CH-positive individuals present in groups predicted to be able to bind the variant (strongly or weakly) vs does not bind it. **b**. Comparison of the fraction of CH-positive individuals present in groups predicted to bind strongly vs bind it weakly or not. **c**. Comparison of the fraction of CH-positive individuals present in groups predicted to bind the variant strongly, weakly vs does not bind it. p-value from chi-squared test. Related to Fig. 2.

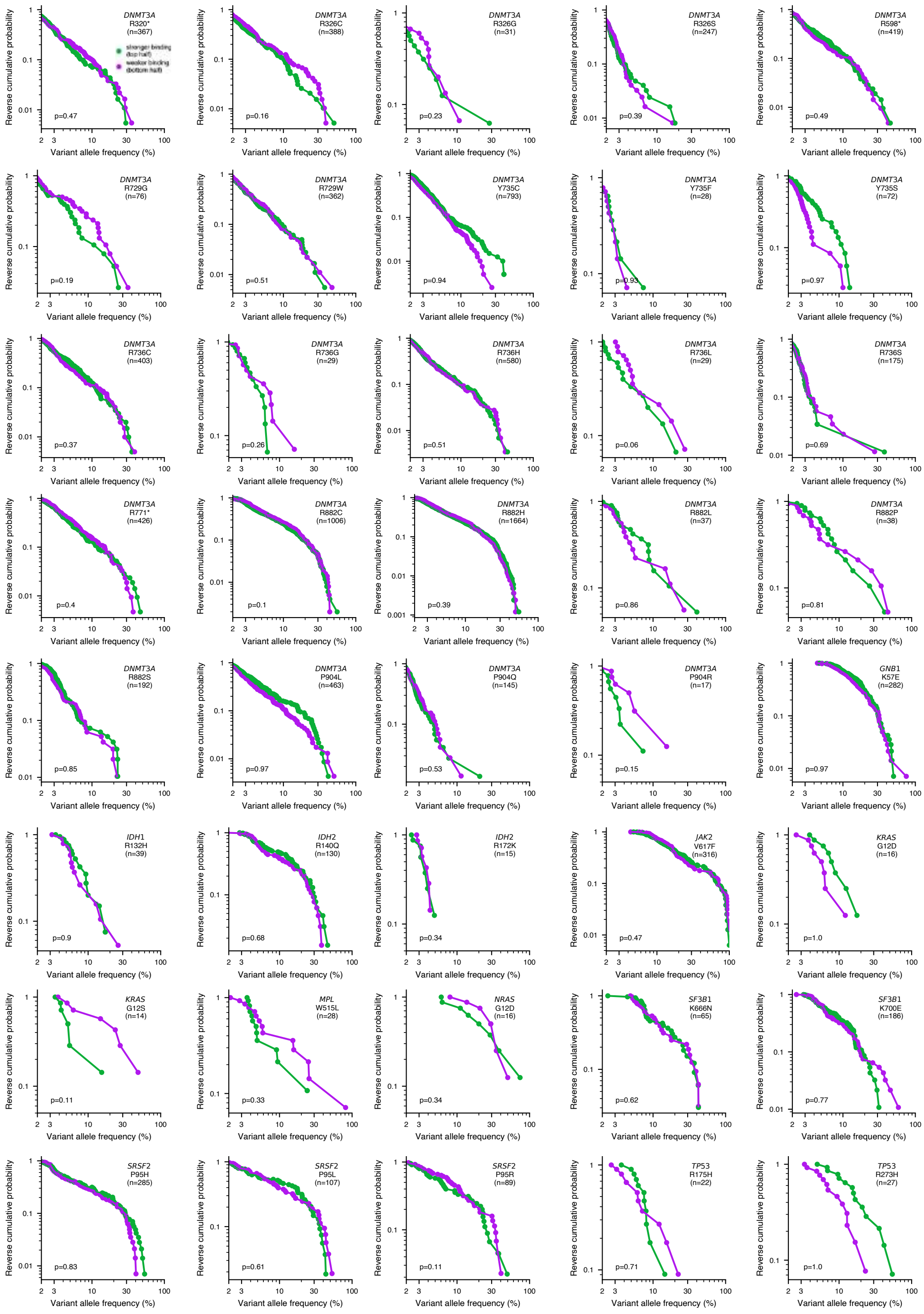

Supplementary Figure 7. Additional analysis based on binding predictions from PRIME2.0. Relationship between predicted MHC-variant binding capacity and clone size in CH-positive individuals for each of the 40 variants examined. p-value from Kolmogorov-Smirnov test (one-sided). Related to Fig. 3.

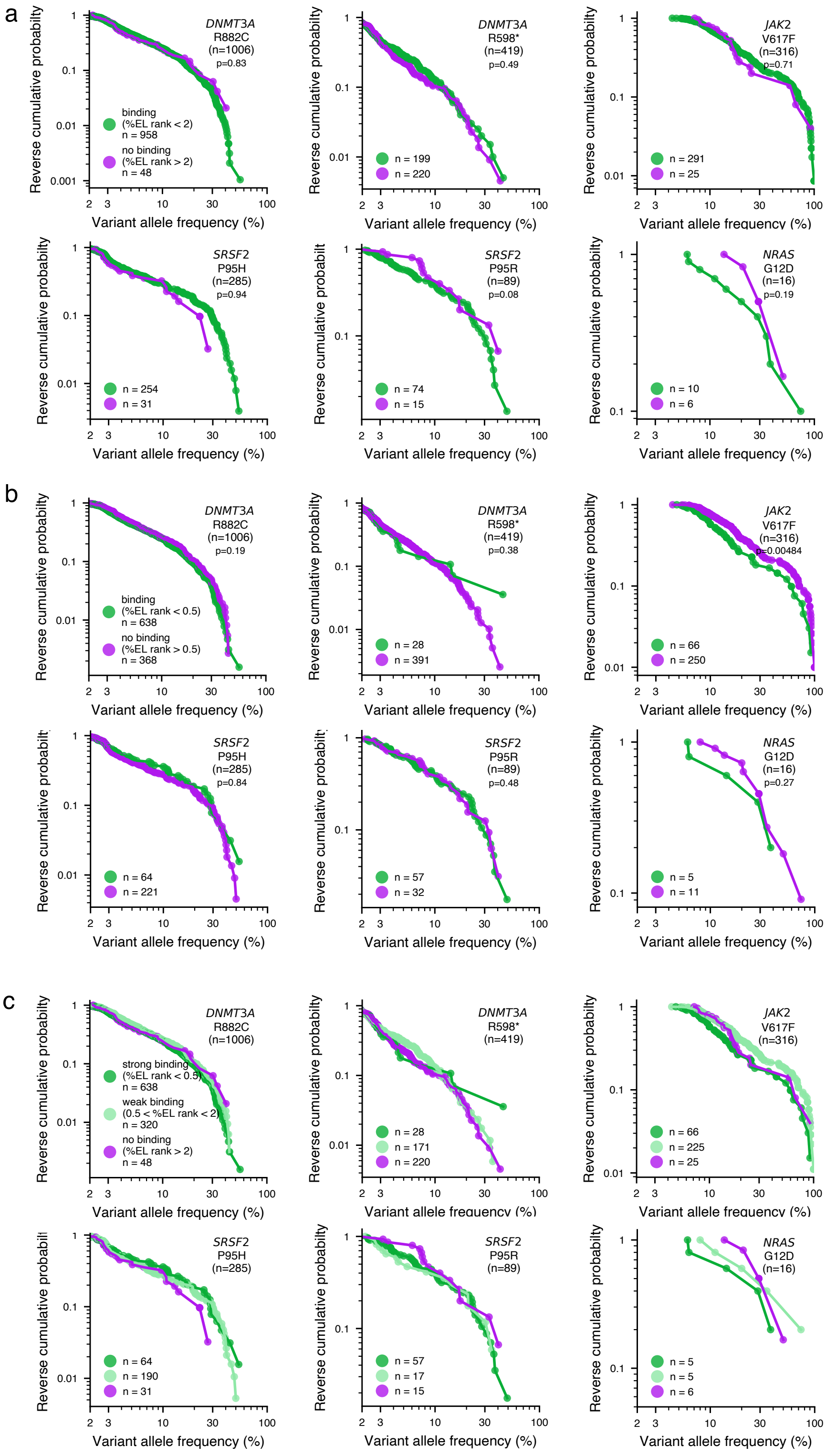

Supplementary Figure 8. Additional analysis based on binding predictions from PRIME2.0. Comparison of reverse cumulative distributions between CH-positive individuals who were classified as binding vs non-binding at different binding thresholds (a – weak binding, b – strong binding, c – weak vs strong binding). p-value from Kolmogorov-Smirnov test (one-sided). Related to Fig. 4.

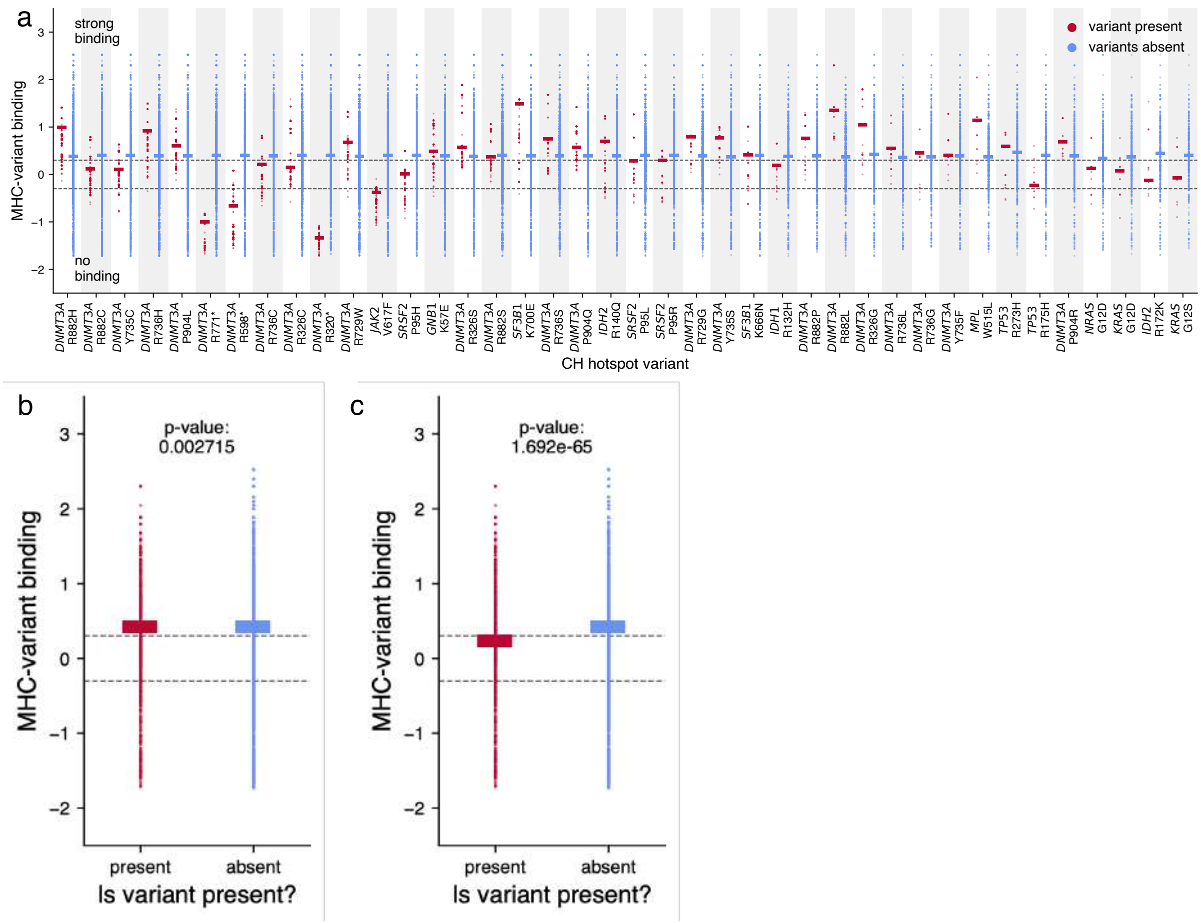

Supplementary Figure 9. a. Binding predictions vary significantly more between variants than between individuals for a given variant. Distribution of scores for variant identified in the individual ('present') and the other 39 possible driver variants examined ('absent'). Analysis carried out on individuals who carry only one, rather than multiple, variants. **b.** Comparison of MHC-variant scores for present vs absent variants when aggregated. We aggregated all scores of variants identified in CH-positive individuals ('present') and compared them to scores of variants they do not carry (39 'absent' variants). All variants (40) included in the analysis. **c**. Comparison of scores of present and absent variants, but with two most common variants, *DNMT3A* R882C and *DNMT3A* R882H (which are also predicted to be bound with high affinity on many MHC alleles), removed from the analysis. p-values from Mann-Whitney U test (two-sided).

Gene	Target variant(s)	chr:position
DNMT3A	R320*	2:25247647
	R326C/G/S	2:25247629
	R598*	2:25244214
	R729G/W	2:25240439
	Y735C/F	2:25240420
	R736H/L	2:25240417
	R736C/G/S	2:25240418
	R771*	2:25240313
	R882C/S	2:25234374
	R882H/L/P	2:25234373
	W860R	2:25235727
	P904L/Q/R	2:25234307
GNB1	K57E	1:1815790
IDH1	R132H	2:208248388
IDH2	R140Q	15:90088702
	R172K	90088606
JAK2	V617F	9:5073770
KIT	D816H/Y	4:54733154
	D816V/F	4:54733155
KRAS	G12D/V	12:25245350
	G12C	12:25245351
MPL	W515L	1:43349338
NRAS	G12D/V	1:114716126
	G12C	1:114716127
SF3B1	K666N	2:197402635
	K700E	2:197402110
SRSF2	P95H/R/L	17:76736877
TP53	R175H	17:7675088
	R273H	17:7673820

Supplementary Table 1. Putative CH drivers targeted in the analysis, originally identified by Watson et al[25]. Genomic positions refer to assembly build 38 (GRCh38).
